# Liquid Chromatography–Electron Capture Negative Ionization–Tandem Mass Spectrometry Detection of Pesticides in a Commercial Formulation

**DOI:** 10.1021/jasms.1c00307

**Published:** 2021-12-13

**Authors:** Achille Cappiello, Veronica Termopoli, Pierangela Palma, Giorgio Famiglini, Mansoor Saeed, Simon Perry, Pablo Navarro

**Affiliations:** † University of Urbino, Department of Pure and Applied Sciences, LC−MS Laboratory, Piazza Rinascimento 6, 61029 Urbino, Italy; ‡ Department of Chemistry, Vancouver Island University, Nanaimo, BC, Canada V9R 5S5; § Jealott’s Hill International Research Centre, Syngenta, Bracknell, Berkshire RG42 6EY, U.K.

## Abstract

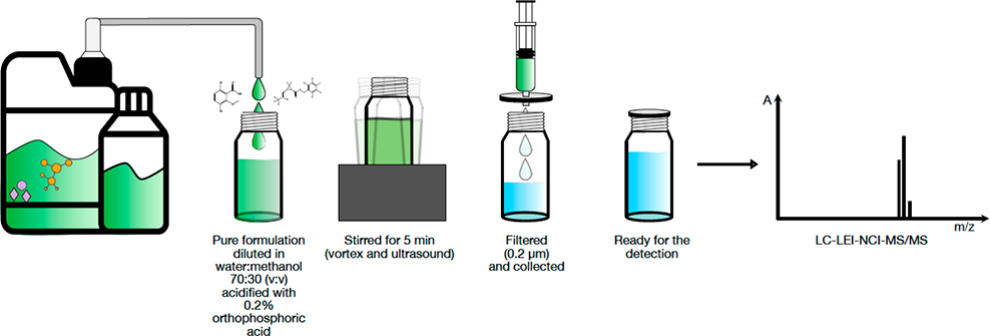

Negative chemical ionization (NCI) and electron-capture negative ionization (ECNI) are gas chromatography–mass spectrometry (GC–MS) techniques that generate negative ions in the gas phase for compounds containing electronegative atoms or functional groups. In ECNI, gas-phase thermal electrons can be transferred to electrophilic substances to produce M^–•^ ions and scarce fragmentation. As a result of the electrophilicity requirements, ECNI is characterized by high-specificity and low background noise, generally lower than EI, offering lower detection limits. The aim of this work is to explore the possibility of extending typical advantages of ECNI to liquid chromatography–mass spectrometry (LC–MS). The LC is combined with the novel liquid-EI (LEI) LC–EIMS interface, the eluent is vaporized and transferred inside a CI source, where it is mixed with methane as a buffer gas. As proof of concept, dicamba and tefluthrin, agrochemicals with herbicidal and insecticidal activity, respectively, were chosen as model compounds and detected together in a commercial formulation. The pesticides have different chemical properties, but both are suitable analytes for ECNI due to the presence of electronegative atoms in the molecules. The influence of the mobile phase and other LC- and MS-operative parameters were methodically evaluated. Part-per-trillion (ppt) detection limits were obtained. Ion abundances were found to be stable with quantitative linear detection, reliable, and reproducible, with no influence from coeluting interfering compounds from the sample matrix.

## Introduction

Negative chemical ionization (NCI) MS shows high selectivity and sensitivity for specific classes of molecules, allowing very low detection limits to be achieved. Ion-forming processes in the presence of a reagent gas can result from different pathways, namely deprotonation, nucleophilic addition, and ion-pair formation. Negative-ion formation can also occur when a molecule captures a low-kinetic energy electron. This pathway is called electron capture negative ionization (ECNI). In ECNI, the primary role of the reagent gas is not to provide a reagent ion but to reduce the electrons’ high energy approaching thermal energy. ECNI spectra are characterized by their limited fragmentation and intense, readily identifiable molecular ions and/or few intense fragment ions.^1−5^ Compared to electron ionization (EI), the chromatogram background noise is minimized, especially in complex samples, because of the absence of interfering impurities from matrix components which do not contain electronegative elements or groups. Consequently, signal-to-noise ratios (S/N) are much higher compared to those obtained with EI, improving limits of detection (LODs) and limits of quantification (LOQs).^6−8^

NCI coupled with gas chromatography mass pectrometry (GC–NCI–MS) has gained significant interest in environmental,^9,10^ biological,^11,12^ and food^4,13,14^ applications due to its high selectivity and specificity for suitable compounds containing electronegative atoms or functional groups. NCI has been used to determine pesticides, such as halogenated pyrethroids and organophosphorus compounds, in complex matrices at trace and ultratrace concentration levels.^15,16^ Húškova et al.^17^ compared NCI and EI performance in the GC–MS detection of endocrine-disrupting compounds (EDCs), reporting significantly higher specificity and selectivity in NCI mode. LODs and LOQs were up to 2 orders of magnitude lower for NCI than EI at analyte concentrations in the parts per thousand range.

The use of tandem MS (MS/MS) allows the selection of characteristic transitions for multiple reaction monitoring (MRM) acquisition to generate characteristic product ions beneficial to implementing structural information for confirmation purposes.^18^ Anagnostopoulos et al.^19^ studied the performance of six different GC–MS methods in detecting pesticide residues in plant matrices. EI and NCI in MS and MS/MS acquisition mode were compared, NCI resulted in higher sensitivity compared to EI in all cases. Raina et al.^20^ confirmed that GC–NCI–MS/MS provided the lowest detection limits, along with the best confirmation, comparing GC–MS and GC–MS/MS with EI and NCI for the analysis of pesticides in atmospheric samples. GC–NCI–MS was found to be beneficial for detecting suitable compounds at ultratrace concentrations and residues analysis for targeted applications. However, acidic and thermolabile pesticides, which account for almost 20% of the present-day pesticides, are not GC-amenable and require derivatization^21−24^ prior to analysis. This step is needed to achieve better separation, vaporization, higher sensitivity, or addition of electrophilic groups.^25^ Furthermore, in aqueous matrices, GC–MS requires time-consuming sample preparation steps with the potential risk of compound losses.

LC–MS has become the standard analytical approach in analyzing a broad range of polar, nonpersistent pesticides enabling multiresidues methods for up to 500 compounds in the same chromatographic analysis.^26,27^ However, LC–MS ionization modes, such as electrospray (ESI) or atmospheric pressure chemical ionization (APCI), are more prone to matrix effects compared to EI, leading to quantification drawbacks.^28−30^ In addition, low polarity analytes, such as pyrethroids, and those with relatively high polarity and low molecular mass, such as acidic pesticides, do not efficiently ionize in LC–MS using negative ESI.^31,32^

Given these drawbacks, an LC–MS system equipped with an EI source can address conventional GC–MS and LC–MS constraints. The liquid electron ionization (LEI) interface is an innovative approach to successfully couple a liquid flow rate from an LC system with an EI-based MS, converting the LC eluate in the gas phase before entering the EI source.^33,34^ LEI has demonstrated the capability to limit matrix interferences even in complex samples, allowing the simultaneous detection of GC-amenable and LC-amenable compounds in the same chromatographic run.^35^ Other LC–MS EI-based interfaces have been proposed by Mondello’s and Amirav’s groups, confirming this approach as a powerful complementary tool in LC–MS applications.^36−40^

Since the LEI interface is coupled with a conventional EI-based MS, it allows operation with different ionization modes, such as EI or chemical ionization (CI). The type of ion source chosen (EI or CI) depends on the analyte’s properties and the type of information required. The LEI interface can benefit from this dual ion source setup. As demonstrated, LEI’s LC mobile phase flow rate is converted in a gas phase before entering the ion source. This mobile phase vapor can act as a proton donor and promote [M + H]^+^ formation when needed.

Vandergrift et al. exploited the LEI interface coupled with condensed phased membrane introduction mass spectrometry (CP-MIMS) with a conventional CI source, demonstrating that the acceptor phase solvent can provide suitable reagent species in the positive ion mode (PCI) for the analysis of dialkyl phthalates and polycyclic aromatic hydrocarbons in dust and soils, respectively.^41,42^

Herein, we present LC–LEI–MS in ECNI mode using methane as a buffer gas to determine dicamba and tefluthrin in single analysis, as model compounds, and added at an ultratrace level in a commercial pesticide formulation (CF) without sample preparation, only dilution, pH adjustment, and filtration.

The simultaneous detection of these two pesticides is challenging due to their different physical–chemical properties. Dicamba is routinely analyzed with ESI-LC–MS, but its extraction from aqueous matrices is challenging due to its high polarity and high-water solubility.^31^ On the contrary, tefluthrin, a nonpolar compound, is scarcely ionized with ESI and typically analyzed with GC–MS. CF is considered to be a very complex matrix because, together with active ingredients, it may contain many other additives, such as solvents, dispersants, and surfactants. Using other MS approaches, this type of matrix would require sample preparation steps to extract the compounds of interest.^43^ Instead, LC–LEI–MS/MS enables analysis of dicamba and tefluthrin simultaneously in the same chromatographic separation without sample preparation.

To the best of our knowledge, this is the first ECNI application in LC–MS using two model compounds with opposite physicochemical properties in a commercially available formulation without derivatization and sample preparation.

## Materials and Methods

### Standards and Reagents

LC–MS grade acetonitrile (ACN) and methanol (MeOH) were purchased from VWR, part of Avantor (Milan, Italy). Ultrapure water was obtained with a Direct-Q 3 UV system from Millipore Corp. (Merck, Milan, Italy). Orthophosphoric acid (PA, 85%), formic acid (FA), and trifluoroacetic acid (TFA) were purchased from Merck (Milan, Italy). Standards of dicamba and tefluthrin (purity >99%) and a CF were provided by Syngenta, Ltd. (Bracknell, UK). Stock solutions of the two pesticides were prepared gravimetrically at a concentration of 2 mg/mL in MeOH and stored at 4 °C. Working standard solutions were prepared volumetrically as combined suites at concentrations of 50, 250, 500, 2500, 5000, and 10000 ng/mL in MeOH.

### Preparation of Standard Solutions for Method Validation

*Step 1*: 150 mg of the original CF was weighed and diluted in 30 mL of a mixture of 70:30 water/MeOH (v/v) and acidified with 0.2% PA (pH ≫2) to obtain a diluted CF of 5 mg/mL. This diluted CF solution was vigorously vortexed for 5 min and divided into 30 1 mL aliquots for method validation purposes.

*Step 2*: 1 mL aliquots of diluted CF were fortified with 10 μL of working standard solutions of dicamba and tefluthrin to obtain the following concentrations: 0.5, 2.5, 5, 25, 50, and 100 ng/mL. Each concentration was prepared in triplicate. Those fortified diluted CF aliquots were filtered using PTFE and 0.2 μm of disposable filters (Econofilter, 5185-5834, Agilent Technologies, Milan, Italy), stored in 1.5 mL glass vials (Agilent Technologies, Milan, Italy), and used for calibration experiments.

*Step 3*: 1 mL aliquots of 70:30 water/MeOH (v/v) acidified with 0.2% PA, without CF, were fortified with 10 μL of working standard solutions, as described in step 2, for calibration experiments and evaluation of matrix effects.

*Step 4*: Three 1 mL aliquots of diluted CF were fortified at concentrations of 0.5, 2.5, and 25 ng/mL for repeatability experiments at low, medium, and high concentration levels. Each concentration was prepared in triplicate.

*Step 5*: Three 1 mL aliquots of diluted CF were fortified at 0.05, 0.1, and 0.2 ng/mL for evaluation of LODs and LOQs.

All measurements were carried out in triplicate, and the relative standard deviation was calculated. LODs and LOQs were calculated as the minimum concentration with a signal-to-noise ratio (S/N) equal to or higher than 3 and 10, respectively.

### LC–LEI–MS/MS Apparatus

A triple-quadrupole Agilent 7010 B QQQ mass spectrometer (Agilent Technologies, Inc., Santa Clara, CA) was equipped with an LEI interface and operating in NCI mode. A detailed description of the interface is reported elsewhere.^33,34^ The vaporization microchannel (VMC) temperature was set at 260 °C, whereas ion source and quadrupole temperatures were set at 150 °C. The ion-source temperature was kept at 150 °C to provide low-energy electrons.^44,45^ Methane (grade 6.0, purity >99.9%) was used as the reagent gas (Nippon Gases, Italy) and introduced into the ion source at 40% (∼2 mL/min). Data acquisitions were carried out in full scan, product ion scan, and MRM modes. The chromatographic separations were performed with an Agilent Infinity II UHPLC system (Agilent Technologies Inc., Santa Clara, CA) using a Phenomenex C18 XB column 1.7 μm particle size (2.1 mm × 150 mm) (Phenomenex, Italy) in gradient elution from 100% solvent A (97:3 water/ACN, v/v) to 100% solvent B (ACN) in 15 min. A and B were acidified with 0.2% PA. The flow rate was set at 100 μL/min with a passive postcolumn splitter to decrease the flow rate to 0.5 μL/min before entering the LEI interface (split ratio 1:200). A detailed description of the postcolumn passive splitter setup is reported elsewhere.^33^ The injection volume was 20 μL. Physicochemical properties, full scan acquisition parameters, precursor and product ions, and optimized collision energy of dicamba and tefluthrin are listed in Table S-1. Optimization of the MRM method for the two pesticides was performed using a two-compound mixture standard solution in 70:30 water/MeOH (v/v) acidified with 0.2% PA at a concentration of 200 ng/mL. Fragmentation of the selected precursor ions was performed by collision-induced dissociation (CID) using nitrogen (purity >99%) at 1.5 mL/min (Air Liquide, Italy). A solvent delay of 6 min was included in all MRM analyses to prevent filament damage. Instrument tuning was carried out once a week using perfluoro-5,8-dimethyl-3,6,9-trioxidodecane (PFDTD) as a reference compound, monitoring its characteristic ions (*m*/*z* 185, 351, and 449). A check tune was performed daily. No mobile phase was admitted into the ion source during these procedures. Agilent Mass Hunter software (version B.08.00) was employed for instrument control and data acquisition processing.

## Results and Discussion

**Figure 1:**
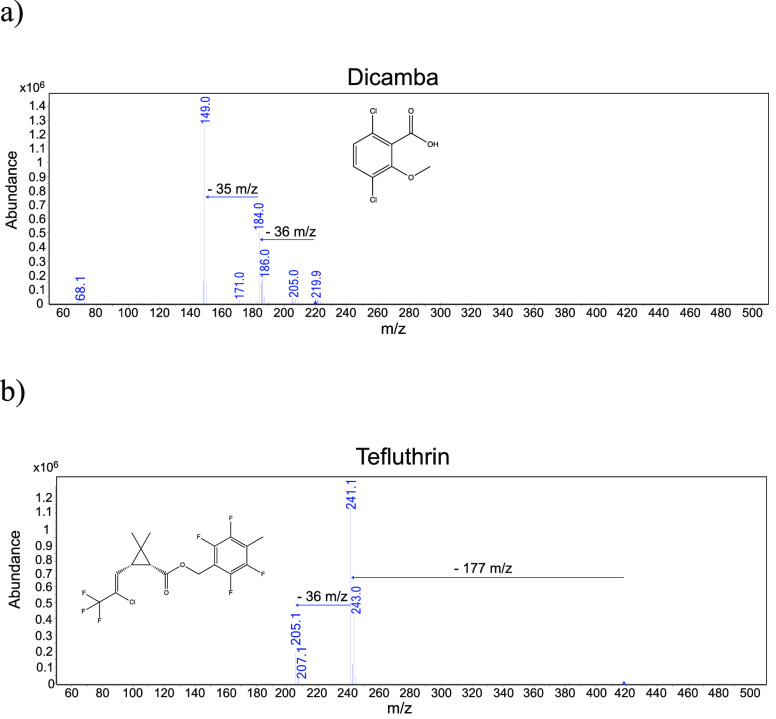
Full-scan spectra of (a) dicamba and (b) tefluthrin in LC–LEI–MS in ECNI mode.

The initial experiment was dedicated to acquiring the full scan spectra of dicamba and tefluthrin recorded for the first time in LC–LEI–MS in negative-ion mode. The full-scan spectra were obtained by injecting 20 μL of a two-compound standard mixture at 500 ng/mL in 70:30 water/MeOH (v/v) and are reported in Figure 1a,b.

After a careful evaluation of the two spectra, ion formation likely occurs under ECNI following two different pathways:^44^

(1) resonance electron capture, in which molecular radical anions, M^–•^, are formed by capture of low energy electrons (0–2 eV):
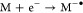
pathway 1
(2) dissociative electron capture in which fragment ions are generated by the capture of 0–15 eV electrons:
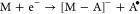
pathway 2

As shown in Figure 1a, dicamba’s mass spectrum is characterized by the presence of a low-abundance molecular radical anion at *m*/*z* 220 and a few fragment ions (*m*/*z* 184 and 149). Therefore, both pathways likely occur in the ionization process. On the contrary, in the spectrum of tefluthrin (Figure 1b) the molecular radical anion is not observed but only two intense fragment ions at *m*/*z* 241 and 205. It is reasonable to conclude that in the case of tefluthrin this occurs via the dissociative electron capture pathway.

### LC–LEI–MS/MS Optimization

#### MS/MS Parameter Optimization

In NCI and ECNI, buffer gases, such as methane, isobutane, or ammonia, are fundamental to provide reagent ions in the first case and decelerate 70 eV electrons generated in the ion source by the filament, obtaining the thermal electrons, in the second case. Therefore, the nature and pressure of the selected buffer gas, amount of sample introduced, and ion source contaminations play a crucial role in the ionization process.

In this work, a mobile phase containing water, acetonitrile, and modifiers is continuously introduced in the CI source. Thus, mobile phase composition, VMC temperature, and methane percentage as a buffer gas were investigated in detail to assess the optimal ionization conditions before progressing to actual matrix experiments.

The MS/MS parameters were optimized using the same two-compound standard solution mixture in 70:30 water/MeOH (v/v) at 500 ng/mL. The precursor ions were selected as the most abundant peaks of each spectrum. Different collision energies (from 5 to 15 eV) were investigated to promote fragmentation. For each compound, the collision energies that yielded the highest signal intensity in precursor–product ion transitions were selected to set up the MRM acquisition method. Because of the limited fragmentation obtained, only two transitions from each precursor ion were used. The most intense transition was chosen as the quantitative transition (Q), whereas the second one was selected as the qualitative transition (*q*), following the ion ratio criteria established by Commission Decision 2002/657/EC.^46^ As identification and confirmation criteria, retention times and *Q*/*q* ratios were considered for both compounds, as reported in Table S-1.

#### Mobile-Phase Composition

Unlike GC–MS, in LC–NCI–MS, oxygenated species present in the mobile phase (i.e., water) introduced inside the ion source can capture low-energy electrons, affecting the ionization process efficiency. Moreover, common acidic modifiers carrying electron-acceptor groups, such as TFA, can also capture low-energy electrons, thus suppressing the ionization efficiency. Due to the acid nature of dicamba, an acid mobile phase (pH 1–3) is required to avoid dissociation and help with the peak shape in reversed phase LC. Tefluthrin is not affected by the mobile phase pH. Therefore, to analyze them together, the mobile phase and the solvent in which they are dissolved must contain an acid modifier.

The influence of three different acidic modifiers in the ionization performance was evaluated by adding FA, TFA, and PA to solvents A and B at the following percentages to obtain pH ≫2: FA 1% in both solvents; TFA 0.050% in solvent A, 0.025% in solvent B; PA 0.2% in both solvents. The signal intensity of the Q transition of dicamba and tefluthrin was monitored in five consecutive injections at 10 ng/mL in 70:30 water/MeOH (v/v). The standard pesticide mixture was divided in three aliquots acidified with FA 1%, TFA 0.050%, and PA 0.2%, respectively. The results are shown in Figure S-1. The highest signal intensity for both compounds was achieved using PA at 0.2% in both solvents. Due to its neutral-basic nature, tefluthrin seems to be negatively influenced only by TFA, which competes for the interaction with the available low-energy electrons, significantly decreasing the signal. FA (p*K*_a_ = 3.75) and PA (p*K*_a_1 = 2.14) do not interact with low-energy electrons; nevertheless, they play a significant role in preventing dicamba (p*K*_a_= 1.87) dissociation. The use of PA improves dicamba sensitivity as it is stronger than FA. TFA (p*K*_a_ = −0.25) is the strongest of the three modifiers, more suitable to prevent dicamba dissociation; however, it competes with dicamba for low-energy electrons needed for the ionization, resulting in a worse sensitivity. Overall, PA was considered to provide the best sensitivity and the most consistent peak area for both compounds.

#### Optimization of VMC Temperature and Methane Percentage in the Ion Source

**Figure 2:**
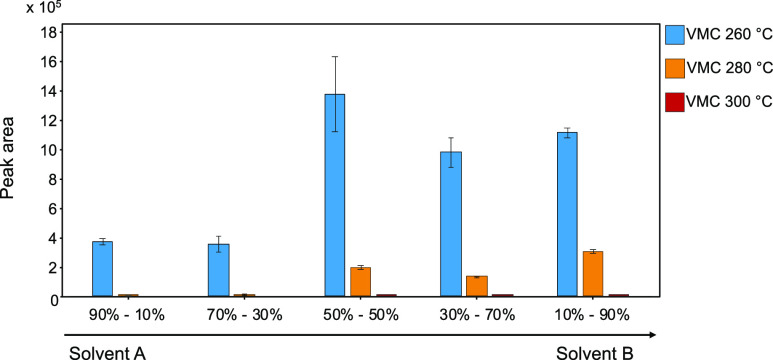
Integrated peak area values of dicamba Q transitions at different VMC temperatures and mobile-phase compositions.

Particular attention was given to selecting the VMC working temperature because of the thermal lability of the dicamba. The VMC temperature is commonly set between 350 and 400 °C to ensure rapid and efficient vaporization of the eluate from the LC system. However, these temperatures are too high and provoke thermal degradation of dicamba, with erroneous ions displayed in the spectrum. VMC temperatures ranging from 260 to 300 °C and mobile-phase compositions varying from 90% solvent A to 90% solvent B were evaluated. These experiments were carried out in flow injection analysis (FIA) using an external manual injector with an internal loop of 0.01 μL (Vici, Switzerland) after the passive splitter. Five consecutive injections at a 5 μg/mL concentration in methanol were acquired for standard deviation calculations. Figure 2 shows the abundance of the Q transition area at each VMC temperature, with varying mobile phase composition. It is evident that the peak area abundance of dicamba decreases as VMC temperature increases and that a high-water percentage affects the ionization efficiency. The highest yield was obtained at 260 °C with a percentage of solvent B equal to or higher than 50%.

**Figure 3:**
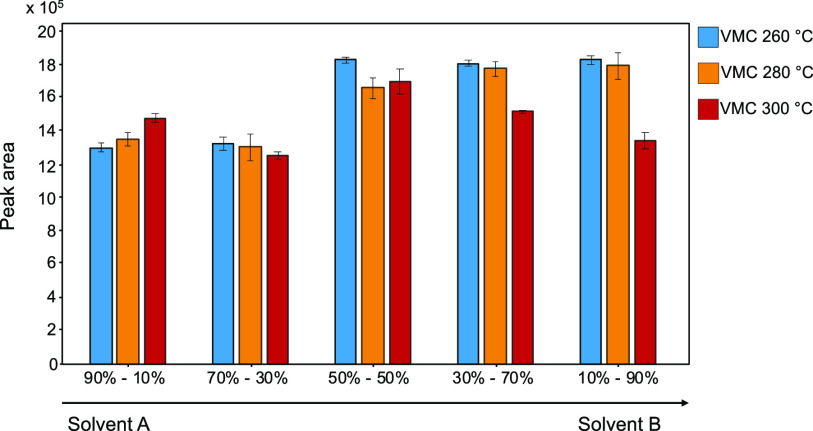
Integrated peak area values of tefluthrin Q transitions at different VMC temperatures and mobile-phase compositions.

The same set of experiments was carried out to evaluate tefluthrin response at different experimental conditions. The results obtained, shown in Figure 3, demonstrate that tefluthrin is not severely affected by VMC temperature or by mobile phase composition; therefore, VMC temperature was set at 260 °C, optimal for dicamba.

Temperatures lower than 260 °C do not promote an efficient vaporization process.

Both compounds were found to elute in gradient conditions at a high percentage of ACN (80% for dicamba and 100% for tefluthrin), thus overcoming the low response observed at a high percentage of water.

**Figure 4:**
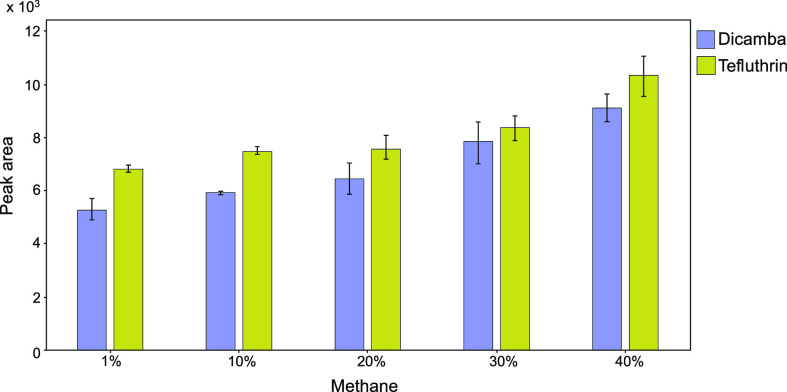
Influence of different methane percentages on Q transitions on integrated peak areas values of the two pesticides.

The presence of methane as buffer gas in the ion source is fundamental to promote ECNI; therefore, the amount of methane requires careful evaluation. The Q transition intensities of the two compounds at 10 ng/mL in 70:30 water/MeOH (v/v) with PA 0.2% (v/v) were monitored at the following percentages of methane: 1, 10, 20, 30, and 40 as reported in Figure 4. Percentages higher than 40% may damage the filament and the high-vacuum pump according to the manufacturer recommendations for GC–MS operation. As shown in Figure 4, 40% of methane yielded the highest peak area values for both compounds.

#### System Performance

Using the optimized parameters, the system performance was evaluated considering LODs and LOQs, linearity, intra- and interday repeatability, and matrix effects (ME). The values obtained, reported in Table 1, were achieved by injecting 20 μL of the diluted CF fortified solutions (see the Material and Methods) considering the less intense transition (*q*) for both compounds. Method robustness was assessed considering interday and intraday repeatability, carried out in fortified diluted CF solutions at three different concentrations: 0.5, 2.5, and 25 ng/mL. All concentrations were injected five times for six consecutive days, as reported in the Material and Methods. As shown in Table 1, the results demonstrate excellent repeatability with RSD < 9% for intraday and <12% for interday experiments.

**Table 1:** Calibration Data, Detection and Quantification Limits, and Matrix Effects in Solvent and Diluted CF

compd	LOD (ng/mL)	LOQ (ng/mL)	linearity range (ng/mL)	equation in solvent	*R*^2^	equation in CF	*R*^2^	ME (%) (slope CF/slope standard) × 100
dicamba	0.08	0.3	0.5–100	*y* = 473.51 *x* + 800.97	0.9987	*y* = 486.33 *x* + 715.7	0.9978	103
tefluthrin	0.05	0.2	0.5–100	*y* = 1578 *x* – 1235	0.9999	*y* = 1604.4 *x* – 594.27	0.9974	102

	intraday repeatability in diluted CF (avg area of *q* transition, five injections, % RSD)			interday repeatability in diluted CF (avg area of q transition, 6 days, five injections, % RSD)		
	low (0.5 ng/mL)	medium (2.5 ng/mL)	high (25 ng/mL)	low (0.5 ng/mL)	medium (2.5 ng/mL)	high (25 ng/mL)
dicamba	119 (6)	1729 (5)	12351 (4)	115 (12)	1476 (10)	12568 (8)
tefluthrin	330 (4)	2648 (5)	40128 (3)	313 (5)	2886 (8)	40543 (4

The high selectivity of ECNI for electrophilic compounds excludes most matrix interferences, such as additives, resulting in almost absent background noise.

**Figure 5:**
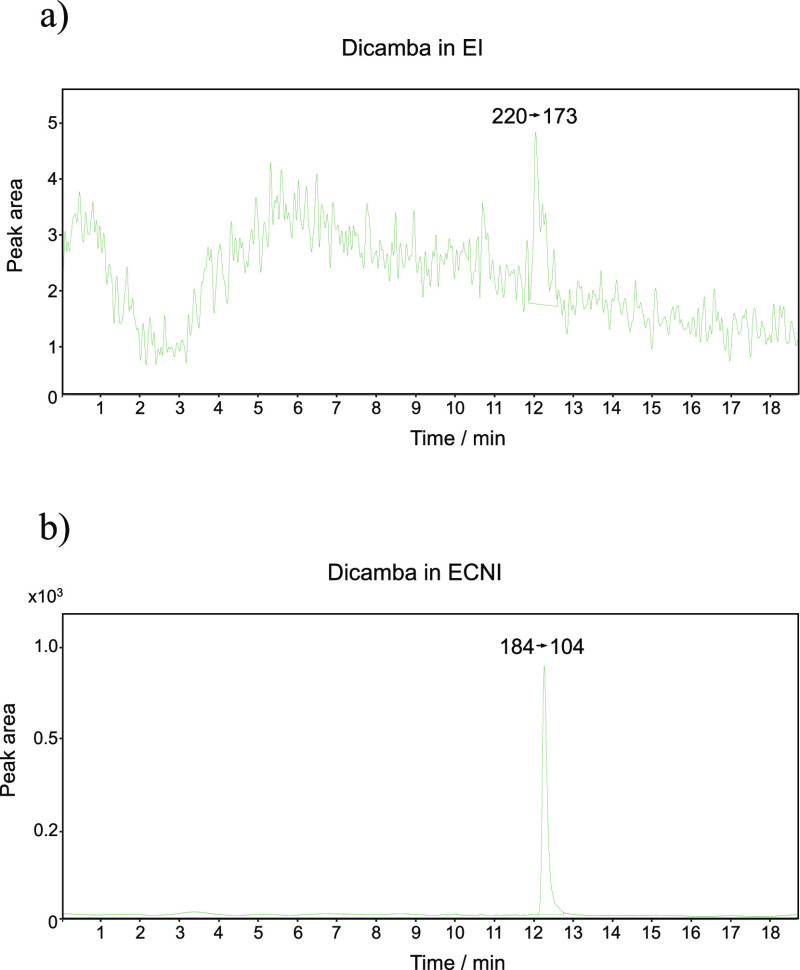
*Q* transition of dicamba in diluted CF at 5 ng/mL with LC–LEI–MS/MS in (a) EI and (b) in ECNI.

This aspect is essential considering two crucial factors: no sample preparation was carried out on the complex matrix (except dilution) and the mobile phase was introduced into the ion source. Very low LODs and LOQs were achieved for both compounds, ranging from 0.05 (tefluthrin) to 0.08 (dicamba) ng/mL and 0.2 (tefluthrin) to 0.3 (dicamba) ng/mL, respectively. This sensitivity is particularly evident in dicamba detection, resulting in a 100-fold lower LOD. As observable in Figure 5, the *Q* transition of dicamba in diluted CF at 5 ng/mL in EI is characterized by high background noise, leading to low detectability and sensitivity, whereas in ECNI signal-to-noise ratio is greatly improved. Tefluthrin gave similar LOD values for both ionization methods. Therefore, in the analysis of dicamba, the LEI interface proved to be more sensitive when coupled with an ECNI source rather than an EI source.

As reported in Table 1, good linearity was achieved with *R*^2^ > 0.9974 for both compounds.

LODs and LOQs data were compared with those reported in the literature (Table 2) and obtained with GC–NCI–MS/MS and LC–MS in similar applications. The sensitivity obtained in this work for these two compounds is comparable with those reported in previous publications, demonstrating the applicability of the proposed method in trace analysis applications. Moreover, it is essential to point out that the method presented does not require any preconcentration steps.

**Table 2:** Applications Involving the Analysis of Dicamba and Tefluthrin in Different Matrices

compd	matrix	preparation method	detection mode	LOD	LOQ	ref
	raw agricultural commodities	milling/high-speed agitation	LC–ESI–MS/MS	0.3 μg/kg	1.0 μg/kg	(31)
dicamba	food	modified QuEChERS	LC–ESI–MS/MS	0.001 mg/kg		(49)
	groundwater	SPE	LC–ESI–MS/MS	0.0003 μg/L	0.0004 μg/L	(50)
	tobacco	QuEChERS	UPLC–ESI–MS/MS	0.117 ng/g	0.390 ng/g	(51)
	soil			0.126 ng/g	0.420 ng/g	
	commercial formulation	dilution/filtration	LC–LEI–NCI–MS/MS	0.08 ng/mL	0.3 ng/mL	this work
tefluthrin	food	vortex/filtration	GC–NCI–MS	0.02–0.06 μg/kg	0.08–0.2 μg/kg	(15)
	water	DLLME	LC–ESI–MS/MS	0.62 μg/L	0.75 μg/L	(32)
	sediment			2.5 ng/g	7.50 ng/g	
	food	homogenization/agitation/centrifugation	GC–NCI–QTOF	0.5 μg/kg	5 μg/kg	(5)
	food	homogenization/blending/homogenization	GC–NCI–MS		1 μg/kg	(7)
	commercial formulation	dilution/filtration	LC–LEI–NCI–MS/MS	0.05 ng/mL	0.2 ng/mL	this work

**Figure 6:**
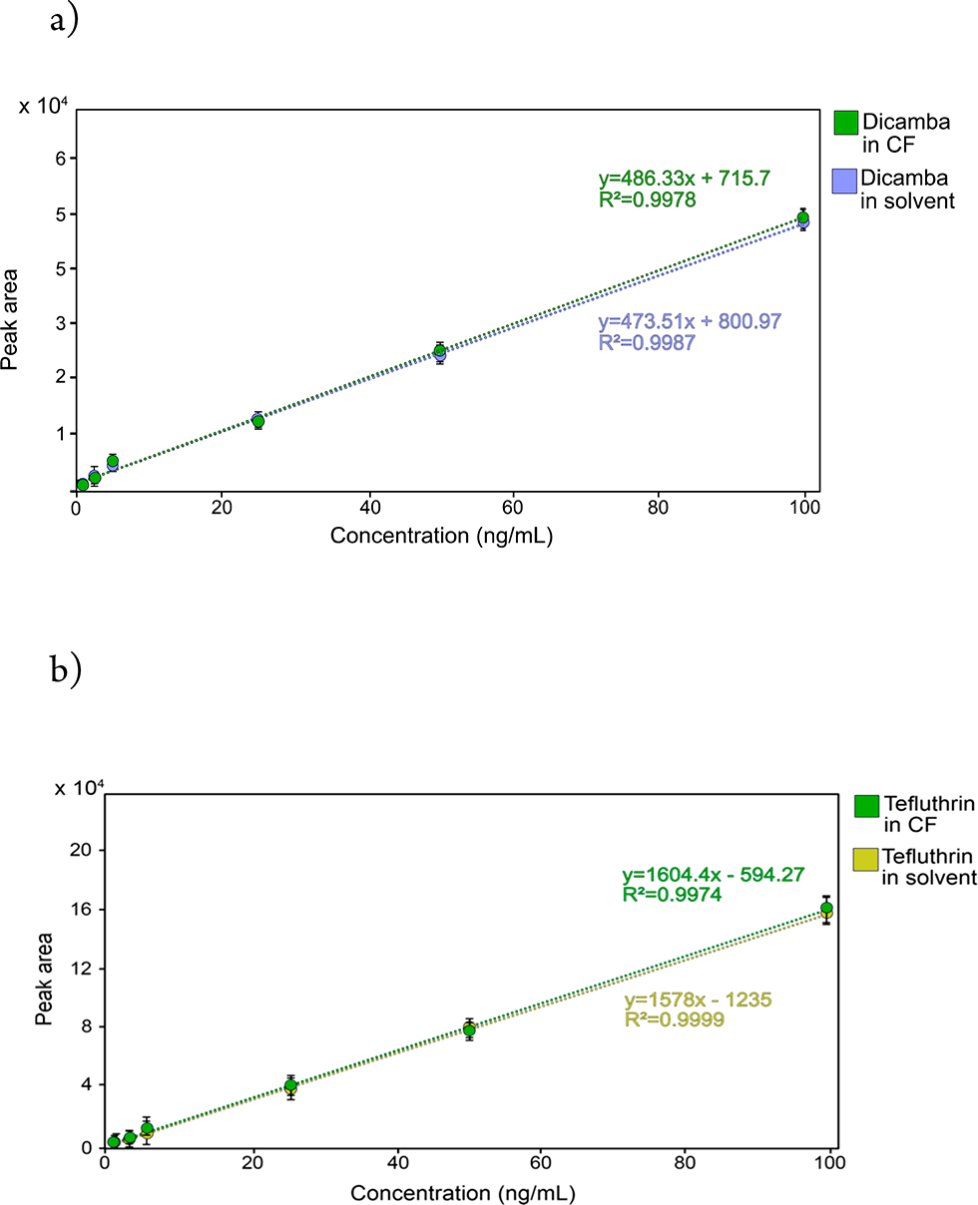
Calibration curves of a two-compound mixture in 70:30 water/MeOH (v/v) with PA 0.2% and in diluted CF: (a) purple dots, dicamba in solvent; green dots dicamba in diluted CF; (b) yellow dots, tefluthrin in solvent; green dots, tefluthrin in diluted CF.

For ME evaluation, the calibration curves of the two pesticides in two-compound standard mixtures were compared with those obtained in fortified diluted CF solutions. In ideal conditions, the total absence of ME is recognized when the two curves are overlapping.^47,48^ The comparison of the two slopes in solvent and diluted CF, shown in Figure 6, indicates the total absence of ME, allowing an accurate quantification in this complex matrix. To emphasize this result, MRM profiles of the two-compound mixture at 0.5 ng/mL in 70:30 water/MeOH (v/v) with PA 0.2% and diluted CF at the same concentration were compared, as shown in Figure S-2. No differences in peak areas are noticeable, even in the presence of interferences coming from the matrix that was only diluted and filtered. These results are in accordance with other previous publications^33−35^ indicating that LEI response is scarcely affected by coeluted compounds from the matrix.

With more than 50 analyses per week, great attention was given to the CI source cleaning. Because of its characteristic lens geometry and introduction of methane and matrix, this source is more prone to contamination than EI. Therefore, careful cleaning twice a month was crucial to maintain consistent instrumental performance, which is also the typical cleaning protocol required for GC–NCI operation.

## Conclusions

NCI plays an essential role in GC–MS for its unparalleled sensitivity and specificity for electrophilic compounds. In this work, the applicability of ECNI with an LC–LEI–MS/MS interface is demonstrated for the first time. The ion source, designed by the manufacturer for gas intake, was not altered before using a liquid phase. The system operation was not influenced by the liquid phase inlet composed of a mixture of two solvents, a modifier, and a complex commercial formulation. The LEI interface permitted the efficient vaporization and transfer of the eluate from the LC system to the ion source.

The method was applied to the determination of a mixture of dicamba and tefluthrin as model compounds. These two pesticides, with opposite physicochemical properties, were determined in a commercial pesticide formulation. No sample preparation steps were needed, only an appropriate matrix dilution with 70:30 water:/MeOH (v/v) acidified with PA 0.2% (v/v).

High sensitivity, with LODs at the ppt level, was obtained, demonstrating that ECNI allows a 100-fold sensitivity increase compared to LC–LEI–MS. The matrix complexity did not influence the ionization efficiency, and no signal suppression or enhancement was observed. The experimental setup was simple, and the system robustness was demonstrated. Although CI in GC–MS is considered less repeatable than EI, the data obtained in LC–MS presented was found to demonstrate similar performance, despite the greater complexity of the mobile phase.

Hence, NCI in general and ECNI with LC–MS represents a simple and attractive alternative for electrophilic compounds in complex matrices, opening the way to new perspectives in many applications. Future work will be directed to other compatible pesticides and direct analysis without the use of a chromatographic column.

## Supporting Information Available

Additional information on compounds physicochemical properties, modifier percentages, and MS acquisition parameters (PDF)
