# Supplementary material for: Liquid Chromatography–Electron Capture Negative Ionization–Tandem Mass Spectrometry Detection of Pesticides in a Commercial Formulation
Source: J Am Soc Mass Spectrom. 2021 Dec 13;33(1):141–8. doi: 10.1021/jasms.1c00307 (PMC8739837; doi:10.1021/jasms.1c00307)
Supplement: Supplementary file 1 — js1c00307_si_001.pdf [file js1c00307_si_001.pdf]

Supporting Information

Liquid Chromatography-Electron Capture Negative Ionization Tandem-Mass Spectrometry Detection of Pesticides in a Commercial Formulation

Authors:

Achille Cappiello[1,2]*, Veronica Termopoli[1], Pierangela Palma[1,2], Giorgio Famiglini[1], Mansoor Saeed[3], Simon Perry[3], Pablo Navarro[3]

Author Affiliations:

[1] University of Urbino, Department of Pure and Applied Sciences, LC-MS Laboratory, Piazza Rinascimento 6 – 61029 Urbino, Italy

[2] Department of Chemistry, Vancouver Island University, Nanaimo, BC, Canada V9R 5S5

[3] Jealott's Hill International Research Centre, Syngenta, Bracknell, Berkshire, RG42 6EY, UK

*Corresponding author: achille.cappiello@uniurb.it

Contents:

Additional information on compounds physicochemical properties, modifier percentages, and MS acquisition parameters.

Table S-1. Physicochemical properties of dicamba and tefluthrin, full scan MS, and MRM acquisition parameters.

Figure S-1. Effects of formic acid (FA), trifluoroacetic acid (TFA) and orthophosphoric acid (PA, 85%) on integrated peak areas of the two-compound mixture of dicamba and tefluthrin at 10 ng/mL in 70:30. See text for modifiers percentages.

Figure S-2. MRM profiles of the Q transitions of dicamba and tefluthrin at 2.5 ng/mL. a) Two-compound mixture in 70:30 water:MeOH (v:v) PA 0.2%; b) Two-compound mixture in diluted CF.

Table S-1. Physicochemical properties of dicamba and tefluthrin, full scan MS, and MRM acquisition parameters.

Compound	MW	Vapor pressure*	log Kow*	pKa*
**Dicamba**	219.7	9.24 x 10^{-6} mm Hg at 25 °C	2.21	1.87
**Tefluthrin**	418.1	6.00 x 10^{-5} mm Hg at 20 °C	6.4 at 20 °C	≥ 9
	**Acquisition parameters**			
	**Full scan**			
	**Acquisition range (m/z)**	**Scan time (ms)**	**Threshold**	**Gain**
	55-500	700	10	0.1
	**Multiple Reaction Monitoring (MRM)**			
		**Precursor**	**Product**	**Collision energy (eV)**
	**Dicamba**	(Q)[a]184	104	10
		(q)[b] 184	148	5
	**Tefluthrin**	(Q)[a] 241	205	15
		(q)[b] 241	135	5

*PubChem database
[a]Q (quantitative transition)
[b]q (qualitative transition)

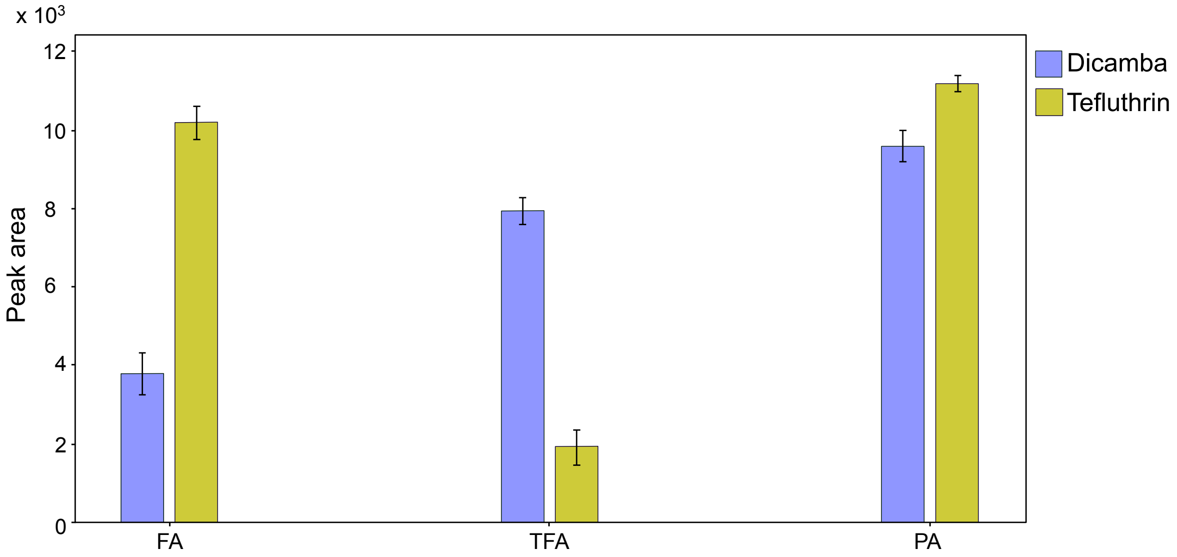

Figure S-1. Effects of formic acid (FA), trifluoroacetic acid (TFA) and orthophosphoric acid (PA) on integrated peak areas of the two-compound mixture of dicamba and tefluthrin at 10 ng/mL in 70:30 water:MeOH (v:v). Modifiers percentages: FA 1%; TFA 0.050%; and PA 0.2%.

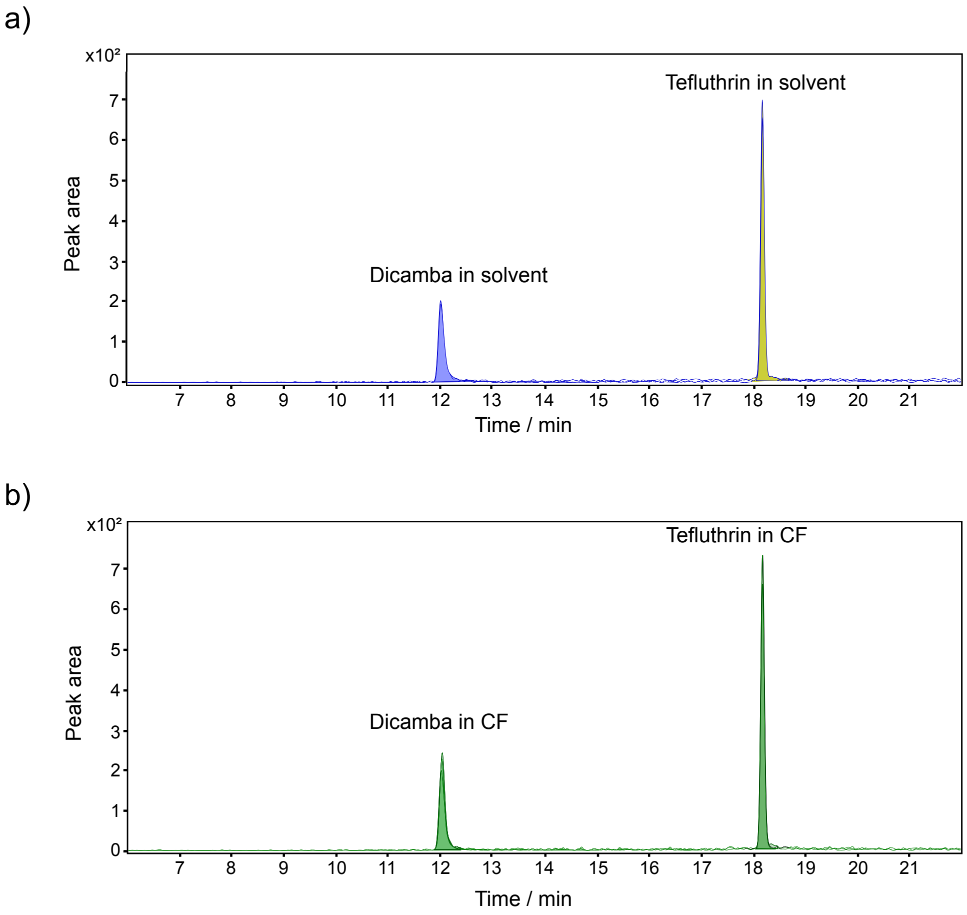

Figure S-2. MRM profiles of the Q transitions of dicamba and tefluthrin at 2.5 ng/mL. a) Two-compound mixture in 70:30 water:MeOH (v:v) PA 0.2%; b) Two-compound mixture in diluted CF.
